# The Use of Complementary and Alternative Treatments in Adolescent Obesity: A Narrative Review

**DOI:** 10.3390/ijerph22020281

**Published:** 2025-02-14

**Authors:** Mahesh Shrestha, Ann Harris, Teresa Bailey, Urvi Savant, Dilip R. Patel

**Affiliations:** 1 Department of Pediatric and Adolescent Medicine, Homer Stryker MD School of Medicine, Western Michigan University, Kalamazoo, MI 49008, USA; mahesh.shrestha@wmed.edu; 2 Department of Medical Library, Homer Stryker MD School of Medicine, Western Michigan University, Kalamazoo, MI 49008, USA; ann.harris@wmed.edu; 3 College of Pharmacy, Ferris State University, Big Rapids, MI 49307, USA; teresabailey@ferris.edu; 4 Homer Stryker School of Medicine, Western Michigan University, Kalamazoo, MI 49007, USA; urvi.savant@wmed.edu

**Keywords:** adolescent obesity, complementary and alternative treatment, body mass index

## Abstract

Adolescent obesity is a growing global health problem all around the world. We reviewed the use of complementary and alternative medicine (CAM) for adolescent obesity, examining improvements in BMI or any metabolic indices of obesity. We performed a PubMed and Scopus search for articles on CAM treatments in adolescents aged 12–17 years, and included all studies with subjects in that age range. Out of 226 PubMed articles and 14 Scopus articles, 28 articles from PubMed and 1 article from Scopus fit our criteria. Most CAM studies that showed some improvement in BMI were acupuncture- or yoga-based. Yoga-based interventions showed a BMI reduction of 1–2 points, which is similar to results achieved in studies based on physical activity and Orlistat, a weak anti-obesity medication; meanwhile, acupuncture-based studies showed a slightly higher BMI reduction of 2–4 points, similar to that achieved with Liraglutide, a GLP-1 agonist that is a good anti-obesity medication. Herbs and supplements showed improvement in metabolic markers of obesity. Stress interventions in mind–body interventions, music skip-rope exercise, and creative drama in physical activity-based interventions also showed improvement in BMI. Although many of the studies reviewed were RCTs, the small sample size of those RCTs is a limiting factor. There may be a role for investigating this topic in larger populations to generate more effective conclusions.

## 1. Introduction and Objectives

Pediatric obesity is a global problem in countries with varying income levels [1,2,3]. The prevalence of pediatric obesity has more than doubled since 1990; 390 million children and adolescents aged 5–19 years were overweight or obese in 2022 [1]. Obesity is defined by the World Health Organization (WHO) as a weight greater than three standard deviations above the WHO Growth Reference Median in children under 5 years, or two standard deviations above the WHO Growth Reference Median in children and adolescents between 5 and 19 years of age [1].

Obesity is a chronic disease with a multifactorial etiology [1,3]. Structural barriers to a healthy lifestyle, such as inadequate access to nutritious foods, a lack of safe locations for physical activity, and a lack of consistent healthcare access, contribute to children’s control [1,3,4]. Exposure to high-calorie snacks and drinks, sedentary behavior [1,3,4], excessive use of screens [5,6], and poor support for self-regulating behaviors further exacerbate weight gain [4]. Obesity is associated with many medical conditions, such as type 2 diabetes mellitus, cardiovascular disease, decreased bone health, endocrine abnormalities, increased risk of cancer [1,2], and autoimmune conditions associated with chronic inflammation [2,3,6]. It also has strong psychosocial consequences for children; stigma, bullying, and discrimination can adversely affect school performance, mental well-being, and quality of life [1,3,4]. Obesity in childhood is associated with a 500% increased risk of obesity in adulthood [4]; therefore, early and effective medical treatment for pediatric obesity is paramount for improving lifelong health.

Current medical treatment guidelines for pediatric obesity center on behavioral interventions [3,4]. Dietary education is the mainstay of treatment, focusing on measures such as increased intake of fruits and vegetables and reduced intake of high-calorie snacks and drinks [2,3,4]. Other lifestyle interventions include encouragement of moderate physical activity and decreased sedentary behaviors [3,4]. Pharmacotherapies such as Orlistat and, more recently, Liraglutide and other GLP-1 agonists have been approved by the United States Food and Drug Administration (US FDA) for adolescents older than 12 years [2,7]. Gastric bypass surgery can also be an effective intervention for certain adolescents, although the risk of remission of diabetes or mental health disorders after surgery is higher in this age group than in adults [2,3].

Complementary and alternative medical treatments are often of interest as a holistic approach to address obesity and its comorbidities [6]. Adolescents and adults may be more willing to try CAM treatments, like yoga, caffeine/coffee/tea, or other herbals, with which they may be more familiar and which they may find safer and easier to try out than newer anti-obesity medications or bariatric surgeries. A quick review of CAM therapies in obesity shows that most studies have been conducted on adults [6,8], and there is a gap in studies on children and adolescents. A variety of herbal supplements, such as *Camellia sinensis*, *Hoodia gordonii*, *and Cynanchum auriculatum*, have been proposed to aid in weight loss by augmenting energy metabolism, binding fat in the intestines for faster excretion, and other unknown mechanisms [6,8]. Other available treatments include acupuncture and noninvasive body contouring (cryolipolysis and laser therapy) [6,8]. However, the literature evaluating the safety and efficacy of these treatments offers conflicting results. We purposely chose to review adolescent obesity for several reasons. Due to adolescents being closer in age to adults, we believe that we may be able to review more studies. Adolescents tend to have more issues with body shape image, such as eating disorders and obesity, which will incentivize them to participate in obesity studies. Parents may be hesitant to try some CAM therapies, such as unknown herbs and supplements, with smaller children, but may be willing to enroll adolescents. We also wanted to include studies on other age groups, such as children and adults, if they included adolescents in their studies.

In this narrative review, we aimed to synthesize the available body of evidence regarding the use of complementary and alternative treatments as therapies for adolescent obesity; examine their efficacy in reducing BMI or other obesity indices, including metabolic or physical parameters; and highlight areas that would be beneficial for further exploration.

## 2. Materials and Methods

We performed a literature review of the use of complementary and alternative medicine (CAM) in adolescent obesity by searching the PubMed and Scopus databases for related English-language articles published on 5 April 2024.

We searched PubMed using the following search terms: (“Complementary Therapies”[Mesh] OR “complementary therap*”[tiab] OR “complementary medicine”[tiab] OR “alternative therap*”[tiab] OR “alternative medicine”[tiab]) AND (“Obesity”[Mesh] OR “Obesity/diet therapy”[Mesh] OR “Obesity/drug therapy”[Mesh] OR “Obesity/therapy”[Mesh] OR obesity[tiab]) AND (“Adolescent”[Mesh] OR adolescent[tiab] OR teen[tiab]); this resulted in 226 records. In Scopus, a title/abstract search for (“alternative medicine” OR “alternative therap*” OR “complementary medicine” OR “complementary therap*”) AND obesity AND (adolescent OR teen) returned 14 results.

All observational studies, RCTs, systematic reviews, and meta-analyses were considered. The age range for inclusion was between 12 and 17 years; however, other studies that included these ages were also included. Although the effects primarily examined were improvements in obesity parameters (such as BMI), in addition, weight, body fat, and metabolic aspects of obesity (such as improvements in lipid profile and glucose levels) were also included.

**Figure 1:**
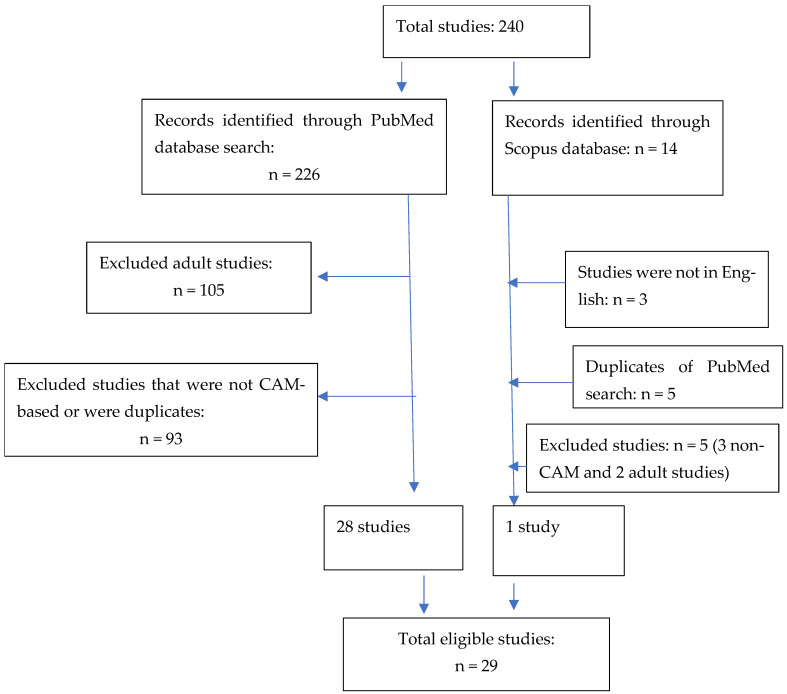
Study flow diagram.

Studies were excluded if the participants were outside of the adolescent age range (≥18 years). Non-CAM and non-English-language studies were also excluded. Of the 226 PubMed records, 105 were excluded because of the age of the participants (≥18 years). Further examination of each study revealed that 93 studies were not CAM studies, leaving 28 studies that met our inclusion criteria. Of the fourteen Scopus records, five studies were excluded because three were non-CAM studies and two were adult studies. Three non-English-language studies and five duplicate records were excluded, leaving one study that met our inclusion criteria. In total, 29 studies were included in this review (Figure 1).

Data were independently extracted by our librarian and co-author using standardized data extraction from 2 main scientific publishing standards, PubMed and Scopus. The main author analyzed the abstracts of the search results to determine full-text eligibility based on our inclusion criteria, and 29 full-text articles were selected. Of the included articles, 16 studies were randomized controlled trials; 6 studies were observational studies; 2 articles were reviews, including 1 systematic review; 2 articles were intervention studies, but not RCTs; and the remaining studies comprised of 1 interview, 1 discussion, and 1 meta-analysis. The selected studies were reviewed by 2 authors, and conflicting findings were discussed; discrepancies were resolved through consensus.

## 3. Results

The initial search for CAM treatments for adolescent obesity yielded 240 results, and 29 studies met the inclusion criteria (Figure 1).The studies have been discussed below and summarized in Table 1.

### 3.1. Acupuncture

The National Center of Complementary and Integrative Health defines acupuncture as a technique wherein practitioners insert fine needles into the skin to treat health problems. The needles may be manipulated manually or stimulated with small electrical currents (electroacupuncture) [35]. Two studies used acupuncture with different modalities to treat obesity in adolescents. Quan et al. [9] performed a systematic review and meta-analysis of 15 randomized control trials (RCTs) involving 1288 overweight/obese children and teenagers between 7 and 16 years in China and Korea, and found improved metabolic indicators with the use of acupuncture, with improvements in BMI, body weight, and serum leptin. Twelve studies showed that the acupuncture group had greater improvements in BMI than the control group, with a standardized mean value (SMD) of −0.45 (95% CI: −0.69, −0.21), and nine trials investigated body weight and found that the efficacy of weight loss in the acupuncture group was better than that in the control group, with an SMD of −0.56 (95% CI: −1.01, −0.10). Limitations of the study were the low number of participants in the RCTs, most of which were from China, and were less representative of other regions. Hong Zhang et al. [10] evaluated acupuncture therapy in 10 healthy obese children and adolescents between the ages of 10 and 13 years daily for a month, and observed statistically significant improvements in body mass index (BMI) and in visceral abdominal tissue using magnetic resonance imaging (MRI) scans. It was found that 1 month of acupuncture therapy reduced BMI by 3.5% (*p* = 0.005), abdominal visceral adipose tissue (VAT) volume by 16.04% (*p* < 0.0001), abdominal total adipose tissue volume by 10.45% (*p* = 0.001), and the abdominal visceral to subcutaneous fat ratio by 10.59% (*p* = 0.007). Decreases in body weight (−2.13%), waist circumference (−1.44%), hip circumference (−0.33%), waist-to-hip ratio (WHR) (−0.99%), abdominal subcutaneous adipose tissue (SAT) volume (−5.63%), and intrahepatic triglyceride (IHTG) content (−9.03%) were also observed, although these were not significant (*p* > 0.05). Limitations were the small study size, but it must be noted that this study studied abdominal visceral fat using MRI, which is difficult to perform in a large study population. MRI studies are the gold standard for assessing actual fat loss compared to BMI, so it is a small but important study.

### 3.2. Yoga

Yoga is an ancient Indian practice that emphasizes the attainment of complete health and well-being through various physical postures (*asanas*), breathing techniques (*pranayama*), and meditation [36]. Jain et al. [11] conducted an RCT in 165 obese children (ages 8–15 years) for 18 weeks, by dividing them into three groups: yoga with dietary modification, standard weight management, and control. It was found that yoga with dietary modification was effective in reducing BMI and systolic blood pressure (BP) compared to the control group, and was as effective as standard weight management for BMI reduction. The median reduction in BMI in the standard and yoga arms was similar in pattern [−1.4 (−3.1, −0.5) kg/m^2^ and −1.2 (−2.3, −0.6) kg/m^2^, respectively], while BMI increased by +0.3 (−0.3, 0.1) in the control arm. The limitations of this study were that not all students attended yoga classes, and there was suboptimal parental involvement. Forseth et al. [12] conducted a pilot study with 30 adolescents with obesity aged 8–18 years, and incorporated PHIT (promoting health in teens) yoga into a 12-week pediatric weight management intervention. Fifty-four percent of the children in the PHIT yoga cohort and 65% of the children in the PHIT Kids cohort (without yoga) attended 75% or more intervention sessions. The survey results showed that PHIT yoga is acceptable to both caregivers and children. Among the PHIT yoga completers, three children (50%) lost weight (average decrease in BMI of 0.90  ±  1.20, and BMI of 95% by 7.4%  ±  1.7%). Similarly, five children (50%) in PHIT Kids lost weight (average reduction in BMI of 0.16  ±  0.06 and in BMI95% by 11.12%  ±  6.9%). The limitations of this study were that it was a small pilot project, although incorporating yoga into a weight management program showed good acceptability by racially diverse obese children. In a systematic review by Chia-Liang Dai et al. [13], nine studies, including 289 participants, were evaluated, and the authors found a small but meaningful impact. While a study carried out by Benavides and Caballero in the USA in 2009 [37] on 12 obese Hispanic adolescents aged 8–15 years showed changes in BMI from 26.4 +/− 6.6 to 25.6 +/− 6.2 kg/m^2^ and a mean weight loss of 2 kg with triweekly Astanga yoga for 12 weeks, and a study by Sarvestani et al. in Iran in 2009 [38], with 60 obese female adolescents aged 11–15, saw a BMI reduction of 1.07 vs. 0.24 kg/m^2^ in 30 experimental group individuals with weekly 4 h sessions for 16 weeks vs. 30 control group individuals, other studies by Hainsworth et al. in the USA in 2014 [39] and 2018 [40] did not show any BMI reduction. The limitations were the small number of studies and the lack of follow-up. Kongkhai Mp et al. conducted an RCT on 40 obese adolescents and studied the effect of continuous yoga (*asanas*, *pranayama*, and *surya namaskar*), and found a significant decrease in BMI and body fat mass and an increase in muscle mass in the yoga intervention group at weeks 8 and 12 [14]. The mean BMI at week 12 for the yoga group was 22.02 (mean difference 0.681: 95% CI 0.204–0.630) vs. 23.42 (mean difference 0.058; 95% CI 0.278–0.395), with a *p* value of 0.018.

### 3.3. Herbs, Supplements, and Diet

There is an extensive history of herb and supplement use to treat various ailments. However, our search yielded very few studies on the use of herbs and supplements in adolescents compared with those on their use in adults.

#### 3.3.1. Fish Oil

The incidence of metabolic syndromes, including central obesity, is increasing. Nonalcoholic fatty liver disease (NAFLD) is a direct consequence of metabolic syndrome. Fish oil has been shown to be beneficial in hypertriglyceridemia. Pacifico et al. [15] reviewed observational and intervention studies to examine the beneficial effects of fish oil in the treatment of NAFLD and the decreasing of lipids in metabolic syndrome. Studies in children have shown conflicting results. Pacifico mentions in his review that while studies by Juarez- Lopez et al. found a significant reduction in BMI after a 12-week treatment with n-3 LC-PUFAs (360 mg EPA and 240 mg DHA), studies by Lopez-Alarcon et al. and De Ferranti et al. did not. Another study by Vasickova et al. found a reduction in body weight after a 3-week therapy with DHA and EPA.

#### 3.3.2. Lignan-Rich Diet

A lignan-rich diet based on olive oil, wheat-based bread, and refined wheat is associated with improved health outcomes. Penalvo et al. [16] determined that boys (aged 2–20 years) with the most lignan-rich diets were less likely to be obese in a population of Spaniard children and adolescents. A strong association was found only for boys between dietary lignan intake and the prevalence of obesity, with an odds ratio (highest versus lowest quartile of lignan intake) of 0.34 (95% confidence interval, 0.17–0.70) after adjusting for main confounders, including dietary fiber. This study suggests that choosing healthier food like olive oil and wheat, as found in lignan-rich diets, may be beneficial in preventing obesity, but more studies may be needed to establish the efficacy of a lignan-rich diet.

#### 3.3.3. Jujube (*Ziziphus jujuba*)

Jujubes have long been used in Chinese medicine for sleep disturbances, anxiety, appetite suppression, and digestion [41]. Sabzghabaee et al. [17] conducted a triple-blind RCT in 80 obese adolescents (aged 12–18 years). Eating 5 g of jujube fruit three times a day for one month was associated with significant decreases in total cholesterol (19 ± 37 mg/dL in controls vs. 170 ± 29 mg/dL in cases, *p* = 0.007) and LDL levels (114 ± 38 mg/dL vs. 104 ± 22 mg/dL, *p* = 0.004). This diet-based study showed the potential of jujube consumption to help improve the metabolic effects of obesity.

#### 3.3.4. Purslane (*Portulaca oleracea*)

Purslane has traditionally been used as an antioxidant and an antiatherogenic agent. Sabzghabaee et al. [18] found a statistically significant improvement in total cholesterol (197.19 to 187.05 mg/dL), LDL-C (112.87 to 101.73 mg/dL), and triglycerides (145.86 to 129.59 mg/dL), with *p* ≤ 0.001, in a randomized controlled trial conducted on obese adolescents aged 12–18 years, where subjects ingested capsules containing 500 mg of purslane seeds twice a day for one month.

#### 3.3.5. Taeumjowi-Tang

Yo et al. [19] studied the use of *taeumjowi-tang*, a traditional Korean herbal formula (KH), in 22 subjects (8 girls and 14 boys; average age 11 ± 2.62 years) for 30 days, and found a statistically significant improvement in BMI and a reduction in serum lipids. The short-term effects of KH on obese children were a reduction in BMI (24.34 ± 3.10 to 23.26 ± 3.00 kg/m^2^) and %RBW (34.41 ± 10.90 to 25.94 ± 11.18%, *p* < 0.01). Their total cholesterol also decreased from 195.38 ± 31.39 to 183.25 ± 33.27 mg/dL (*p* < 0.05). KH was not associated with any adverse effects or safety concerns.

#### 3.3.6. Tiankui

Yu et al. [20] compared the use of *tiankui* capsules (a traditional Chinese kidney-nourishing medicine) and concurrent electroacupuncture to the use of electroacupuncture only in 67 obese adolescents and adults (ages 17–39 years). BMI, body weight, waist-to-hip ratio, fasting plasma glucose, and fasting insulin were lower, and adiponectin levels were higher, in the medicine–acupuncture group than in the acupuncture-only group (*p* < 0.01).

In a review of adult CAM therapies used for obesity by Batsis et al. [8], many herbs, such as *Ephedra*, green tea (*Camellia*), chitosan, *Garcinia*, and chocolate/cocoa, were studied; however, children/adolescent studies with these herbs were not found in our PubMed search.

### 3.4. Mind–Body Interventions

Different mind–body interventions have been attempted to improve adolescent obesity, such as self-hypnosis, stress management programs, and slow deep breathing.

#### 3.4.1. Self-Hypnosis

Cohen et al. [21] studied the use of self-hypnosis (relaxation mental imagery) in 505 children and adolescents aged 3–20 years for behavioral encounters, including obesity or bedwetting, and found a resolution rate of 51% for presenting problems, and a significant improvement in symptoms of 32%. Other articles involving self-hypnosis in adolescent obesity include those by A. Owen-Flood [42] and W. Dikel et al. [22], with similar results. Dikel et al. carried out an RCT with 48 children aged 5–15 years and studied self-hypnosis and biofeedback by dividing them into three groups, and found synergy between self-hypnosis and biofeedback. They showed the efficacy of self-hypnosis in the treatment of conditions like enuresis, pain, asthma, and obesity.

#### 3.4.2. Slow Deep Breathing

Calcaterra et al. [23] showed that slow deep breathing in 301 obese children and adolescents aged 9 to 13 years improved blunted baroreflex sensitivity in children with insulin resistance and diabetes, and may have improved participants’ insulin levels. This study also did not study BMI reduction, but it helped to show that slow deep breathing can provide improvements in metabolic complications of obesity, such as diabetes.

#### 3.4.3. Emotional Freedom Techniques

The rate of adolescent obesity has increased concurrently with the rate of eating disorders. Emotional freedom techniques (EFTs) are techniques that stimulate acupressure points by applying pressure, tapping, or rubbing while focusing on situations that represent personal fear or trauma [43]. EFT is a group of exposure therapies with somatic and cognitive elements which may help in reducing weight and food cravings. Stapleton et al. [24] compared 44 students (aged 12 to 18 years) who used emotional freedom techniques to controls for 6 weeks. There was an improved number of healthy drinks consumed from pre- to post-intervention, although it was not significant (*p* = 0.059). However, there was a statistical improvement (*p* < 0.001) in the number of healthy drinks consumed from post-intervention to follow-up. Similar results were seen from post-intervention to follow-up in self-esteem (*p* < 0.001). Improved eating habits and self-esteem were seen in general, which may be helpful in combating obesity.

#### 3.4.4. Mandolean Method

The Mandolean principle focuses on how people eat, rather than on how much, by focusing on slow eating and small portion sizes. Hilton et al. [25] randomized 24 obese (BMI > 95th perentile) adolescents (aged 11–18 years) into standard care for 6 months in an obesity clinic, versus standard care with short-term Mandolean training. Functional MRI data showed that the Mandolean training group had less activation of brain regions associated with food cue reactivity after a glucose-rich meal, and had 22% smaller portion sizes, with no change in food satiety.

#### 3.4.5. Stress Management

Paltoglou et al. [26] conducted six RCTs on mindfulness and stress management in children and adolescents with obesity. There were 112 subjects (aged 8–17.9 years) in the intervention group and 137 subjects in the control group. Subjects underwent interventions such as mindfulness-based stress reduction therapy for 8 weeks (three studies), mindfulness-based group programs for adolescents (one study), and mindful eating programs for 6 and 10 weeks (one study each). The authors found reduced adiposity markers, such as BMI and waist-to-hip ratio, in four out of six studies, suggesting that mindfulness-based interventions can play a role in decreasing BMI. An RCT conducted by Stavroula Stavrou et al. showed that the application of stress management methods resulted in a significant reduction in body mass index (BMI) in the intervention group compared with the control group [ΔBMI = 1.18 vs. 0.10 kg/m^2^ (*p* < 0.001)]. Another study by Patricia Daly et al. showed that the mindful eating intervention (MEI) group had a decrease in BMI of 1.4 kg/m^2^ by week 10, while the control group’s BMI increased by 0.7 kg/m^2^ at the same time. The limitations of the study were that the RCT groups were small, and not all groups showed lower BMI or improved adiposity markers.

### 3.5. Physical Activity-Based Interventions

The AAP recommends at least one hour of daily physical activity [44]. It is important to understand the effectiveness of physical activity-based interventions in BMI reduction.

#### 3.5.1. Music Skip-Rope

Ham et al. [27] conducted an RCT on 75 obese children and adolescents aged 8 to 13 years in Korea, with exercise counseling and participation in music skip-rope as exercise. They found a statistically significant improvement in self-efficacy (*p* = 0.49) and maintenance of BMI at 6 months (*p* > 0.05), whereas the control group showed an increase in BMI (*p* < 0.05). Music skip-rope-based activity could be fun and engaging, and could prove to be a useful tool in fighting obesity.

#### 3.5.2. Dance-Based Group Exergaming

Wagoner et al. [28] performed an RCT on dance-based group exergaming for 10 weeks on 40 obese adolescents aged 12–18 years, and showed a significant increase in self-reported competence in regular exercise and a significant improvement in participants’ relationships with parents. The AAP also promotes video game-based exergaming to help with obesity [44]. Exergaming could be a useful way to encourage physical activity.

#### 3.5.3. Kung Fu

*Kung fu* is a Chinese martial art that requires training, discipline, and practice. Tsang et al. [29] performed an RCT with *kung fu* training versus a placebo exercise on 20 overweight/obese adolescents aged 12 to 18 years for 6 months. The results of this RCT showed improved enjoyment of physical activity that was greater in the *kung fu* participants than in the placebo group.

#### 3.5.4. Creative Drama

Acar et al. [30] conducted an RCT on 76 overweight/obese adolescents (age 12.5 ± 1 years) in Turkey, evaluating five sessions of creative drama. There was a statistically significant improvement in knowledge, attitude, order of meals, and healthy diet and exercise behavior after the intervention (*p* < 0.05). Statistically significant improvements were observed in BMI, body weight, and waist-to-hip ratio (*p* < 0.05).

#### 3.5.5. Respiratory Muscle Endurance Training (RMET)

Obesity can cause hyposomatropism, owing to blunted growth hormone (GH) responses. Adult studies have shown improvement with RMET using a commercially available device, SpiroTiger. Rigamonti et al. [31] evaluated an incremental RMET-to-bodyweight-reduction program in obese adolescents aged 12–17 years. Improvement in body weight (115.3 ± 9.2 kg to 111.5 ± 8.7 kg, *p* < 0.05) was shown, although no improvement was observed in obesity-related hyposomatropism.

#### 3.5.6. RESPeRATE

RESPeRATE is a device that helps to decrease blood pressure through controlled breathing, and has been shown to be an effective tool for high blood pressure in adults [45]. Wojcicki et al. [32] studied the feasibility of RESPeRATE use in obese children and adolescents 6 to 14 years, and found that 90% still enjoyed using the device after 2 months, and 80% stated that they would recommend the device to a friend or relative. This study is useful in looking at ways to reduce blood pressure, which is a complication of severe obesity in adolescents and adults.

### 3.6. Feedback Therapy

Feedback therapy using a feedback device provides immediate visual or auditory feedback that helps to control adverse eating behaviors in obese patients. However, few studies have been conducted on feedback therapy.

#### 3.6.1. Neurofeedback

Neurofeedback is a type of biofeedback that teaches self-control by measuring brain waves and providing feedback signals in audio or video form [46]. Chirita-Emandi et al. [33] studied 34 obese subjects aged 6–18 years. The participants received 20 neurofeedback sessions, along with standard lifestyle measures. A statistically significant reduction in BMI was observed at 6 months (*p* = 0.035).

#### 3.6.2. Mandometer-Based Feedback

Ford et al. [34] evaluated the use of a mandometer, a computerized device that provides real-time feedback to slow down eating and reduce total food intake. A 12-month evaluation, with reassessment at 18 months after intervention, in 106 obese children and adolescents aged 9–17 years revealed statistically significant improvements in BMI, HDL levels, meal size, and mean fat at 18 months (*p* = 0.001), suggesting a role for feedback devices in retraining feeding behavior as an adjunct to lifestyle changes.

### 3.7. Optimal Healing Environment (OHE)

The Samueli Institute coined the term *Optimal Healing Environment* (OHE) in 2004 to describe a healthcare system designed to stimulate and support the inherent healing capacity of patients, families, and their care providers [47]. Freedman et al. [48] focused on the roles and components of OHE that help in managing pediatric obesity: healing intention, healing relationships, health promotion and disease prevention, and healing spaces. They hypothesized a successful model for the delivery of proper obesity care through OHE by looking at all the tools that are helpful in reducing obesity and that make physicians more comfortable in diagnosing and treating obesity.

### 3.8. Guided Imagery (GI)

Changes in diurnal cortical salivary patterns have been associated with poor metabolic outcomes. Weigensberg et al. [49] studied the use of stress reduction GI and lifestyle behavior GI in 232 Latino adolescents aged 14–17 years, and found a small but statistically significant increase in the subjects’ cortical awakening response. They also observed a reduction in perceived stress using a standardized scale, suggesting that GI may help to improve the stress response and metabolic outcomes in obese adolescents.

## 4. Discussion

The total number of CAM studies on adolescents is far lower than the number of studies performed on adults [8]. This may be because adolescents are a relatively small population, ranging from only 13 to 17 years old, as compared to the available adult population, which ranges from 18 to 65 years. Researchers may be hesitant to conduct child- or adolescent-based research with newer, less-validated treatments. They also likely face additional difficulties in obtaining the necessary IRB approval to conduct research on the vulnerable pediatric population. These obstacles are reflected in the fact that there are considerably more studies investigating the use of herbs and supplements in adults, whereas adolescent-based studies are mostly physical activity-based. Green tea, ephedra, caffeine, chitosan, and cocoa-based adult studies are abundant in the literature [8], whereas adolescent-based studies are more limited to physical interventions, such as dance, and yoga, although acupuncture-based studies are the most common study group for all ages [8].

Many CAM studies on adolescents are region-based. Many acupuncture-based studies have been conducted in China and other Asian countries, such as Korea [9,10,35]. As acupuncture therapy originated in China, there is deep local knowledge of the practices upon which new interventions can be based. One trial studied the effects of electroacupuncture therapy, a variation of acupuncture, in conjunction with the herbal medicine *tiankui*, on adolescent obesity [41]. Yoga originated in India, but it has been widely adopted in many countries, including the USA. Although some studies have been conducted by Indian researchers [11,36], other countries have also conducted yoga-based studies [12,13,37]. Other studies on complementary therapies cited in this review span a wide geographic range, and the lignan-based diet study was based in Spain [16], *taeumjowi-tang* (a herbal tea) was studied in Korea, the *tiankui* capsule and *Ziziphus jujuba* fruit studies were based in China, and the purslane seed (*Portulaca oleracea*) study was based in Iran. An emotional freedom technique was studied in Australia, and a separate device study was conducted in the USA. It may be generalized that countries such as China and Korea, where acupuncture is widely used, are predisposed to conduct acupuncture-related studies because of cultural familiarity, and similar inferences could be applied to some herb-based studies. Yoga-based studies may have a wider geographic distribution owing to the widespread adoption of yoga in many countries. However, the number of studies is too small to make any significant association.

Therefore, it is important to consider the power and quality of these studies. Although many of the studies with statistically significant results that we reviewed here were randomized controlled trials, many also had a small participant pool. This could be because some studies were pilot studies, or because of the difficulty in enrolling adolescents in new untested therapies. Some studies based in China and Russia had no English translations available; therefore, they were either excluded or only briefly highlighted. Although there have been some studies on herbs and supplements such as *taeumjowi-tang* and *tiankui* in adolescents, other herbal formulas that have shown promising results in adults have not been meaningfully studied in this age group.

It would also be interesting to compare these CAM therapies with physical activity, anti-obesity medications, and bariatric surgeries in terms of BMI reduction. Jain et al. [11] studied yoga and standard weight management, and the median reduction in BMI in the standard and yoga arms was similar in pattern (1.4 vs. 1.2 kg/m^2^). The modest reduction in BMI by 1–2 points is similar to other studies on physical activity in adolescents by US Preventive Services Task Force (USPSTF) recommendation statement2024 [50] and AAP clinical practice guidelines[44]. Both AAP and USPSTF studies saw higher BMI reductions with high intensity interventions with more than 26 contact hours over 3–12 months. Similar BMI changes have been observed with the use of the oral anti-obesity drug Orlistat though many studies saw 2.5–3% weight loss over use more than a year over placebo [51]. The SMD (standardized mean difference) is a measure used in meta-analyses to quantify the effect size, adjusting for differences in study designs and populations, and is particularly useful when comparing outcomes across different types of interventions. The results of the acupuncture studies are somewhat surprising. The meta-analysis by Quan et al. [9] showed that 12 studies showed that the acupuncture group had greater improvements in BMI SMD, of −0.45, than the control group, and 9 trials investigated body weight and found that the efficacy of weight loss in the acupuncture group was better than in the control group, with an SMD of −0.56. This is similar to the results achieved with the GLP-1 agonist Liraglutide SMD, given as injection daily with a weight reduction of −0.48, which equates to a BMI reduction of 2–4 points [52] though some studies on Liraglutide and Semaglutide, another GLP-1 agonist given weekly injections saw higher weight loss of around 5–6 points [50,53]. It is to be noted in that in the Quan study, the funnel plot suggested publication bias, but the quantitative Egger test (*p* = 0.235) showed no publication bias. As Quan’s meta-analyses were mostly based on studies conducted in China, there is a need for more representation from studies from other countries in order to validate this impressive BMI reduction. Compared to all other methods, bariatric surgery showed the highest BMI reduction of 10–15 points, with an SMD of −1.1 to −1.3 [54,55].

This review does have a few methodological limitations that may influence some of the findings. First, the total number of studies that fitted our inclusion criteria was relatively small, at 29, so we could not compare lots of studies to see better results. There could be a small publication bias with more positive studies, though we have tried to write about the side-effects of herbs where possible, include studies which showed no efficacy etc. in order to mitigate this risk. The quality of the studies varied significantly, with many being small and lacking randomization, although it was positive to see a meta-analysis and many randomized control trials in this review. Narrative reviews also have limitations, as they may introduce subjectivity and bring more qualitative judgment, rather than quantitative judgment. Also, the generalizability of the findings may be limited to more Eastern countries, like China, India, Asian countries, etc., and may not represent a diverse group of adolescents with obesity around the world.

Based on the findings of this review, several directions for future research can be identified. The development of high-quality and randomized controlled trials with large and diverse sample sizes may provide effective CAM therapies for adolescent obesity. Long-term follow-up studies may also be needed to determine the safety and sustainability of these therapies. Comparative studies between complementary treatments, or between complementary medicine, and anti-obesity drugs or lifestyle programs could be helpful to guide treatment decisions in the future.

## 5. Conclusions

Although somewhat limited by sample size, many of the RCTs and observational studies included in this review had statistically significant results, showing that acupuncture, yoga, and physical therapy-based interventions could play an important role in adolescent obesity. Yoga-based interventions showed a modest BMI reduction, similar to those seen in other physical activity-based studies, by 1–2 points. Acupuncture-based studies showed a slightly higher BMI reduction, similar to that seen with Liraglutide, a GLP-1 agonist, although more studies in countries other than China may be needed to observe the same effect. The herbs and supplements mentioned in our review showed improvements in metabolic markers of obesity. Stress interventions in mind–body interventions, and music skip-rope exercise, creative drama, and RMET in physical activity-based interventions, showed improvement in BMI. With medical providers largely acknowledging obesity as a chronic disease, and with the recent US FDA approval of many anti-obesity medications for the treatment of adolescents, options for the treatment of overweight and obesity in young patients are expanding. Therefore, larger studies on herbs, supplements, and other CAM methods are needed to provide a better understanding of alternative treatments for adolescent obesity.

## Figures and Tables

**Table 1:** Summary of CAM therapies for adolescent obesity.

CAM Method	Type of Study	Results
1. Acupuncture		
1.1. Tingwei Quan et al. [9]	Meta-analysis	SMD BMI −0.45, 95% CI −0.69 to −0.21
1.2. Hong Zhang et al. [10]	Direct Intervention	Decreased BMI by 3.5% (*p* = 0.05)
**2. Yoga**		
2.1. Vandana Jain et al. [11]	RCT	Yoga improved BMI and systolic BP
2.2. Bethany Forseth et al. [12]	Intervention study	Yoga improved BMI
2.3. Chia-Liang Dai et al. [13]	Systematic review	Yoga interventions were small and meaningful
2.4. Na Nongkhai M.P. et al. [14]	RCT	Significant decrease in BMI and weight with continuous yoga
**3. Herbs and Supplements**		
3.1. Fish oil: L. Pacifico et al. [15]	Review	Fish oil beneficial in Metabolic syndrome and NAFLD
3.2. Lignan-rich diet: J.L. Penalvo et al. [16]	Direct observation	Higher lignan-based diet associated with less obesity
3.3. Jujuba: Sabzghabaee A.M. et al. [17]	RCT	Jujuba may decrease TC 179 +/− 29 gm/dL (*p* = 0.007)
3.4. Purslane: Sabzghabaee A.M. et al. [18]	RCT	Purslane improved serum lipid profile
3.5. Taemjowi-tang: Junh-Hee Yoo et al. [19]	Direct observation	Improved BMI (*p* < 0.01) and TC (*p* < 0.05)
3.6. Tiankui capsules: Li-qing Yu et al. [20]	RCT	Use of tiankui capsules with electroacupuncture was better for reducing BMI and fasting glucose than electroacupuncture alone (*p* < 0.01)
**4. Mind–Body Interventions**		
4.1. Hypnosis: D.P Cohen et al. [21]	Direct observation	Self-hypnosis helped in solving half of behavioral encounters
4.2 Self-hypnosis with biofeedback: Dikel et al. [22]	RCT	Self-hypnosis helped with obesity
4.3. Slow deep breathing: V. Calcaterra et al. [23]	Observation	Deep breathing may help in insulin resistance
4.4. Emotional freedom: Peta Stapleton et al. [24]	RCT	Helped in eating behaviors
4.5. Mandolean method: Elanor Hinton et al. [25]	RCT	Mandolean training helped with decreasing portion sizes, with no change in post-meal satiety
4.6. Stress interventions: G. Paltoglou et al. [26]	RCT	Decreased BMI and waist-to-hip ratio
**5. Physical Activity-based Interventions**		
5.1. Music skip-rope exercise: Ok Kyung Ham et al. [27]	RCT	Improvement in self-efficacy (*p* = 0.049) and BMI
5.2. Dance-based group exergaming: T.L. Wagoner et al. [28]	RCT	Significant improvement in perceived competence in exercising regularly
5.3. Kung fu: Tracey W. Tsang et al. [29]	RCT	Improved enjoyment of physical activity
5.4. Creative drama: Mukkaddes D. Acar et al. [30]	RCT	Improved knowledge, attitude, and behavior regarding healthy eating, and improved BMI (*p* < 0.05)
5.5. RMET: A.E. Rigamonti et al. [31]	Direct observation	Improved BMI (*p* < 0.05), but no significant change in GH responsiveness
5.6. RESPeRATE: Janet M. Wojcicki et al. [32]	Interview	Of total participants, 90% enjoyed using device at 2 months
**6. Feedback Therapy**		
6.1. Neurofeedback: Adela Chirita-Emandi et al. [33]	RCT	No improvement in BMI (*p* = 0.337)
6.2. Mandometer-based feedback: Anna L Ford et al. [34]	RCT	Improved BMI (*p* < 0.001) and mean body fat (*p* = 0.1), and increased HDL (*p* = 0.043)
**7.** **Optimal Healing Environment**	Discussion	OHE components may be useful in managing obesity
**8.** **Guided** **Imagery**	RCT	Improved cortisol awakening response, which helped to improve metabolic outcomes

SMD = standardized mean difference.

## Data Availability

Data will be available upon request.
